# Supplementary material for: Low-coverage whole-genome sequencing facilitates accurate and cost-effective haplotype reconstruction in complex mouse crosses
Source: Mamm Genome. 2025 Jul 1;36(4):1063–80. doi: 10.1007/s00335-025-10148-6 (PMC12365916; doi:10.1007/s00335-025-10148-6)
Supplement: Supplementary file 1 — Supplementary file1 (docx 1949 KB) [file 335_2025_10148_MOESM1_ESM.docx]

**SUPPLEMENTAL MATERIALS**

| Library Type | RepeatMasker | VISTA Enhancers | Whole-genome |
| --- | --- | --- | --- |
| lcWGS | 2.185 ± 0.195** | 1.924 ± 0.183 | 1.914 ± 0.171 |
| ddRADseq | 13.532 ± 2.786 | 14.692 ± 3.68 | 13.908 ± 3.066 |

**Supplemental Table 1**

Summarized coverage estimates obtained from each of the 48 DO pilot samples at the whole-genome level, as well as filtering on UCSC RepeatMasker and VISTA enhancer element tracks.

** = Statistically significant increase from Whole-genome coverage estimates.

| **Coverage** | **ddRADseq** | **lcWGS** |
| --- | --- | --- |
| Full Coverage | 8.649 ± 1.664 | 10.079 ± 2.819 |
| 0.5X | 10.733 ± 3.62 | 9.832 ± 2.417 |
| 0.1X | 12.773 ± 3.805 | 9.737 ± 1.748 |
| 0.05X | 14.784 ± 6.181 | 12.942 ± 3.526 |
| 0.01X | 62.503 ± 32.247 | 64.897 ± 45.759 |
| 0.005X | 399.758 ± 324.797** | 744.696 ± 672.017** |
| 0.001X | 518.667 ± 1141.184** | 391.137 ± 853.992** |

**Supplemental Table 2**

Average minimum haplotype block length in kilobases detected among all DO pilot samples. Mean minimum haplotype block length detected using GigaMUGA = 57.884 kb ± 31.814 kb.

**Significant difference among library preparation methods.

| Mapped PE Reads | 546.66 ± 116.14 |
| --- | --- |
| hcWGS coverage | 24.28 ± 4.84 |
| 30X coverage (% of genome) | 26% ± 21% |
| Called hcWGS SNPs (million) | 13.46 ± 0.55 |
| Filtered hcWGS SNPs (million) | 5.24 ± 0.25 |
| Evaluated SNPs (million) | 4.38 ± 0.22 |
| lcWGS coverage | 1.16 ± 0.28 |
| Imputed lcWGS SNPs (million) | 30.02 ± 0 |
| lcWGS-hcWGS SNP concordance | 0.95 ± 0.01 |
| lcWGS-array haplotype concordance | 0.97 ± 0 |

**Supplemental Table 3**

Summary metrics of hcWGS experiment (*n* = 10), SNP concordance, and haplotype concordance of lcWGS in the subset of samples subjected to hcWGS

**
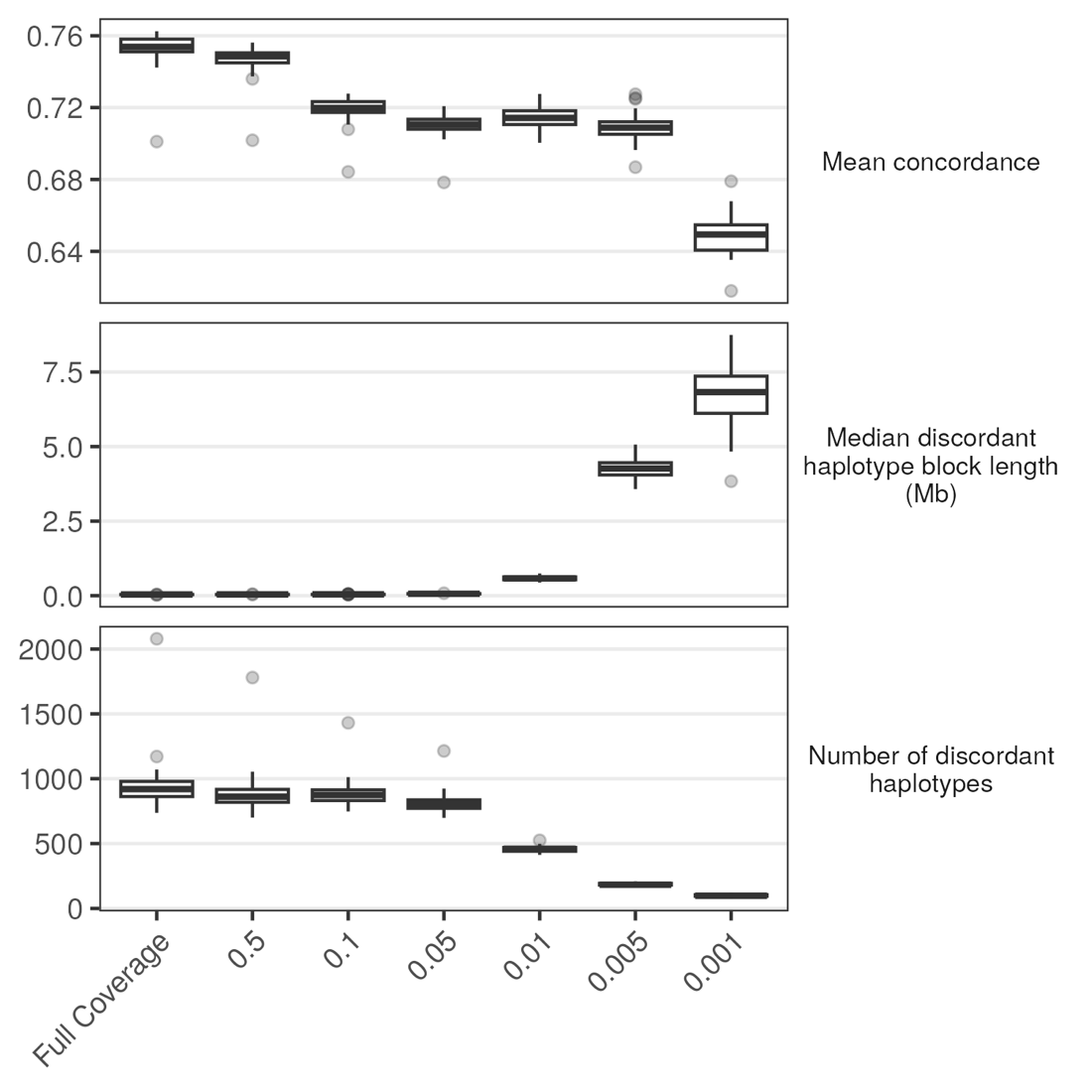
**

**Supplemental Figure 1**

Characteristics of lcWGS haplotype blocks with concordance lower than 0.87 when compared to GigaMUGA haplotype blocks in the same sample at varying coverage specifications.

**
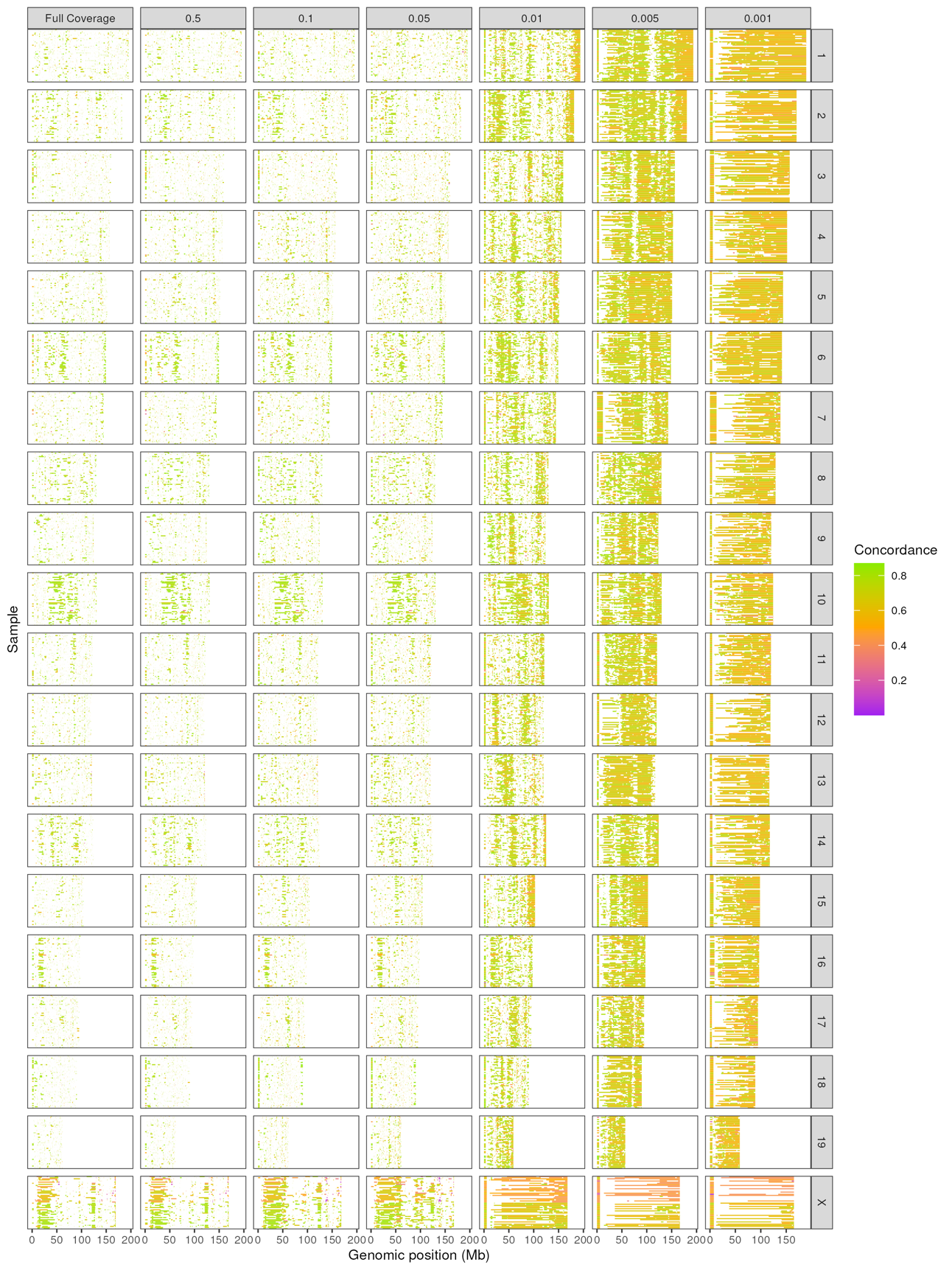
**

**Supplemental Figure 2**

Map of allele probability concordance between lcWGS and GigaMUGA haplotype reconstructions. Each horizontal stripe within each facet represents a region of concordance lower than 0.87 for a given sample, chromosome (vertical facets), and sequencing coverage specification (horizontal facets) combination.


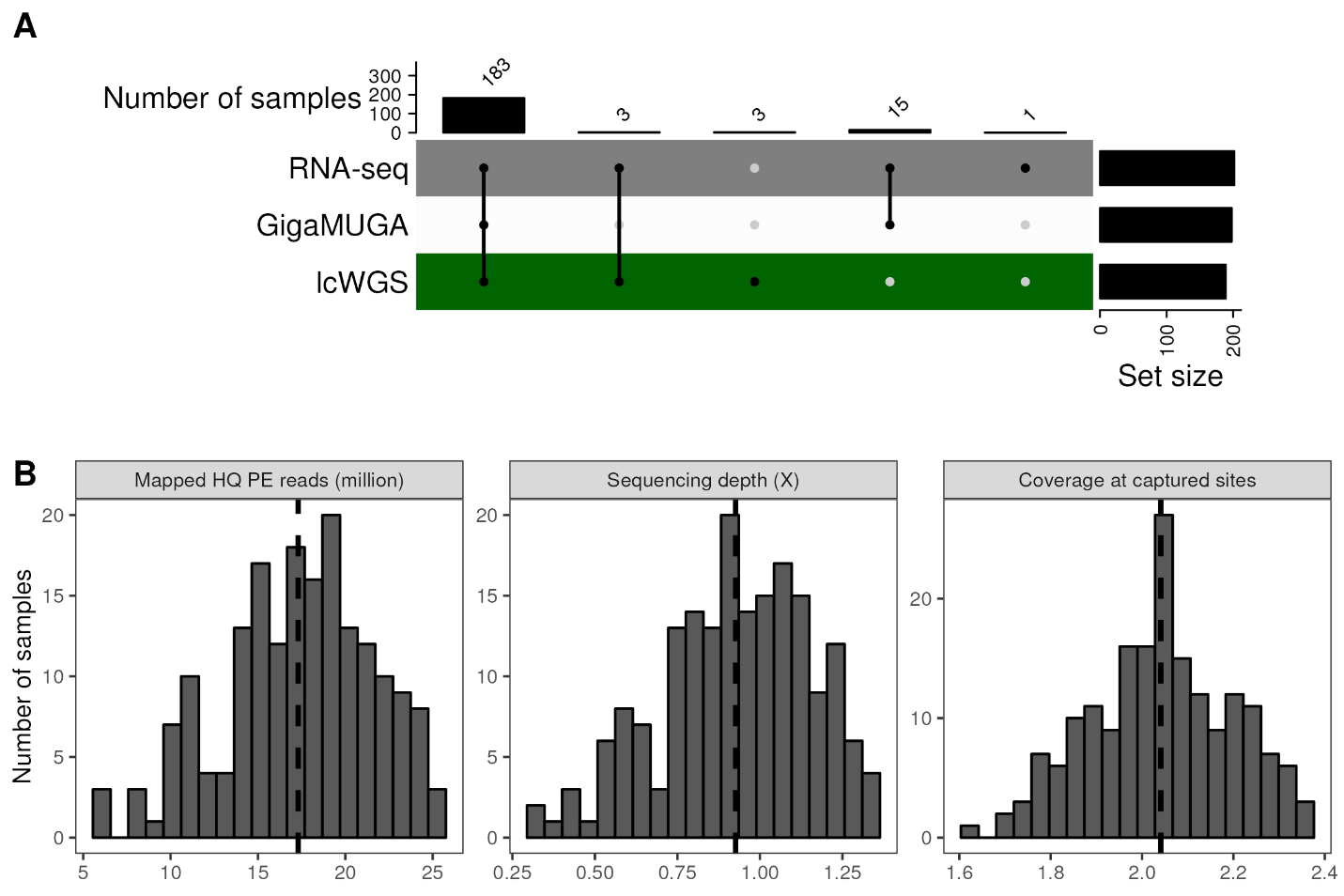


**Supplemental Figure 3**

Overview of sample composition and sequencing metrics of DO embryoid bodies (EBs) used for expression QTL analysis. A) UpSet plot (Gu et al., 2016) of DO EBs and data types obtained. B) Sequencing metrics for 183 DO EBs used for lcWGS.
